# Diffusion and molecular interactions in a methanol/polyimide system probed by coupling *time-resolved* FTIR spectroscopy with gravimetric measurements

**DOI:** 10.3389/fchem.2014.00002

**Published:** 2014-01-30

**Authors:** Pellegrino Musto, Michele Galizia, Pietro La Manna, Marianna Pannico, Giuseppe Mensitieri

**Affiliations:** ^1^Institute of Chemistry and Technology of Polymers, National Research Council of ItalyNaples, Italy; ^2^Department of Chemical, Materials and Industrial Production Engineering, University of Naples Federico IINaples, Italy

**Keywords:** diffusion, FTIR spectroscopy, polyimide, methanol

## Abstract

In this contribution the diffusion of methanol in a commercial polyimide (PMDA-ODA) is studied by coupling gravimetric measurements with *in-situ, time-resolved* FTIR spectroscopy. The spectroscopic data have been treated with two complementary techniques, i.e., difference spectroscopy (DS) and least-squares curve fitting (LSCF). These approaches provided information about the overall diffusivity, the nature of the molecular interactions among the system components and the dynamics of the various molecular species. Additional spectroscopic measurements on thin film samples (about 2 μm) allowed us to identify the interaction site on the polymer backbone and to propose likely structures for the H-bonding aggregates. Molar absorptivity values from a previous literature report allowed us to estimate the population of first-shell and second-shell layers of methanol in the polymer matrix. In terms of diffusion kinetics, the gravimetric and spectroscopic estimates of the diffusion coefficients were found to be in good agreement with each other and with previous literature reports. A Fickian behavior was observed throughout, with diffusivity values markedly affected by the total concentration of sorbed methanol.

## Introduction

Polyimides are high-performance technopolymers characterized by outstanding properties in terms of thermal stability, mechanical performances, high *T_g_* and good resistance to solvents (Bessonov and Zubkov, 1993; Gosh and Mittal, 1996).

These properties make them attractive for numerous applications, among which in microelectronic and opto-electronic devices, or as membranes for separation technologies (Feger et al., 1993; Thompson et al., 1994). Several polyimides have also been employed as polymeric components of high-performance hybrid systems, prepared via the sol-gel route (Mascia, 1995; Hibshman et al., 2003; Musto et al., 2006). The use of diverse polyimide membranes for dehydration of alcohols by pervaporation processes is well documented in the literature (Okamoto et al., 1992; Chen and Martin, 1995). The optimal design of such processes requires a molecular level understanding of the interaction between the penetrant and the polymer matrix. More generally, the theme of H-bonding between low-molecular weight compounds and the polymer substrates within which they diffuse, represents an area of intense research activity, as demonstrated by the increasing number of studies appearing in the literature on the subject. By using solid-state NMR (Jelinski et al., 1985) and *time-resolved* FTIR spectroscopy (Musto et al., 2007, 2012) it has been demonstrated that both water and alcohols are able to form hydrogen bonds with polymers displaying proton acceptor groups on their backbone, and that such an occurrence strongly affects the transport properties and the separation performances of these systems. The occurrence of self-association of the penetrant to form larger molecular aggregates has been also documented for the water/polyimide system (Musto et al., 2007, 2012), but remains a matter of debate for the majority of the investigated systems with water as penetrant, and is an open issue for the case of methanol. In fact, most of the literature studies report only experimental data on methanol diffusion in polyimides, while there is a lack of information about the molecular mechanisms of these processes, and the role played by the different types of H-bonding interactions.

In the present contribution *time-resolved* FTIR measurements have been performed at different relative pressures of methanol vapor to investigate its diffusion into a commercial polyimide. The scope of the study was to characterize the system at the molecular level in terms of number of H-bonding aggregates, their structure and relative population. Gravimetric measurements in the same experimental conditions were also performed to substantiate the spectroscopic results. In terms of diffusion kinetics, a Fickian behavior was observed with diffusivities markedly affected by the total concentration of sorbed methanol.

## Experimental

### Materials

**Scheme 1:**
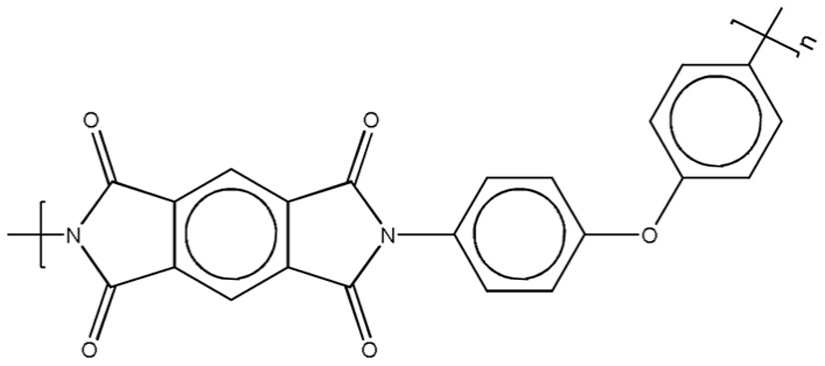
**Molecular structure of the PMDA-ODA polyimide**.

The polyimide precursor used in this study was a polyamic acid, Pyre-ML RK 692 from I.S.T (Indian Orchard, MA). It has molecular weights *M_w_* = 1.0 · 10^5^ g/mol, and *M_n_* = 4.6 · 10^4^ g/mol, and is supplied as a 12 wt% solution in a mixture of N-methyl-2-pyrrolidone (NMP) and xylene (weight ratio 80/20). The polyamic acid is obtained by condensation of pyromellitic dianhydride (PMDA) and oxydianiline (ODA). The molecular structure of the PMDA-ODA polyimide is reported in Scheme 1.

Methanol used for sorption experiments was purchased from *Sigma-Aldrich* (Milano, I) with purity higher 99.6%: it was further purified and degassed through freezing-thawing cycles.

Free standing films of PMDA-ODA, 20–24 μm thick, were obtained by spreading the polyamic acid solution on a clean glass support with a Gardner knife. The nascent films were then dried 1 h at room temperature and 1 h at 80°C, allowing the solvent to evaporate. The castings were then thermally treated in a stepwise manner at 100, 150, 200, 250, and 290°C for 1 h at each temperature. Finally, the films were removed from the glass support by immersion in distilled water at 80°C. Thinner films (2–4 μm) were prepared by a two-step spin-coating process, by using a Chemat KW-4A apparatus (Northridge, CA). Spinning conditions were 12 s at 700 rpm for the first step and 20 s at 1500 rpm for the second step. After removal of the films from their glass support in distilled water at room temperature, they were treated in the same conditions as for the thicker samples.

### FTIR sorption experiments

*Time-resolved* spectra were collected in the transmission mode making use of a vacuum-tight FTIR sorption cell in which a free standing polymer film is exposed to methanol vapor at constant temperature (30°C) and different relative pressures of the penetrant. Full details of the experimental apparatus are reported in Cotugno et al. (2001). Before each sorption measurement, the polymer film was dried overnight under vacuum in the sorption cell to ensure complete removal of absorbed moisture, which was confirmed by the absence of the water bands in the sample spectrum.

The sorption cell was accommodated in the sample compartment of a suitably modified FTIR spectrometer [Spectrum GX from *Perkin-Elmer* (Norwalk, CT)], equipped with a Ge/KBr beam splitter and a wide-band DTGS detector. Instrumental parameters for data collection were set as follows: resolution = 4 cm^−1^; Optical Path Difference (OPD) velocity = 0.5 cm/s; spectral range 4000–600 cm^−1^. Spectra were acquired in the single-beam mode using a dedicated software package for *time-resolved* spectroscopy (*Timebase*, *Perkin-Elmer*).

Differential sorption tests were performed by increasing stepwise the relative pressures of methanol vapor within the range 0–0.6.

### Gravimetric sorption experiments

Methanol sorption experiments were performed at 30°C, using a Quartz Spring Balance equipped with a couple of digital CCD cameras. Experimental sorption isotherms were obtained by increasing stepwise relative pressure of the penetrant (*p*/*p*_0_, *p*_0_ being the penetrant vapor pressure at the experimental temperature). Before each sorption test, the polymer sample was dried overnight under vacuum at the experimental temperature, up to constant weight. Full details about the experimental procedure are given in Cotugno et al. (2005).

### FTIR data analysis

Full absorbance spectra (i.e., polyimide plus absorbed methanol) were obtained using a background collected on the empty cell (i.e., without sample) at the test conditions. The spectra representative of absorbed methanol were obtained by using as background the single-beam spectrum of the cell containing the dry polymer film. The spectrum obtained in this way is equivalent to that resulting from the difference spectroscopy method (subtraction factor, *K* = 1), provided that the sample thickness does not change significantly during the sorption measurement, which was experimentally verified in the present case. The above procedure allows us to eliminate the interference of the polymer spectrum from the methanol peaks located in the 3650–2750 cm^−1^ range [the ν (OH) and ν (CH) vibrations] and at around 1000 cm^−1^ [ν(C-O), vide infra]. To separate the individual components in the case of unresolved bands, a curve fitting algorithm was applied, based on the Levenberg–Marquardt method (Marquardt, 1963; Meier, 2005). The peak functions used for the two components were a log-normal line-shape for the sharp peak at higher frequency and a Gaussian profile for the broader band at lower wavenumbers, which are expressed, respectively, as (Meier, 2005):

(1) $$f(x)=H\exp\text{ ​}\left[\frac{-\ln2}{{\left(\ln\rho \right)}^{2}}\ln^{2}\text{ ​}\left[\frac{\left(x-x_{0})\;(\rho^{2}-1\right)}{w}\right]+1\right]$$

(2) $$f(x)=H\exp\text{ ​}\left[{\left(\frac{x-x_{0}}{w}\right)}^{2}4\ln2\right]$$

where *x*_0_ is the peak position; *H* the peak height; *w* the full-width at half height (FWHH), and ρ the asymmetry index (half width ratio). In order to keep the number of adjustable parameters to a minimum, the baseline and the number of components were fixed, allowing the curve-fitting algorithm to optimize the height, the FWHH and the position of the peaks.

## Results and discussion

### Interpreting absorbance and difference spectra

**Figure 1:**
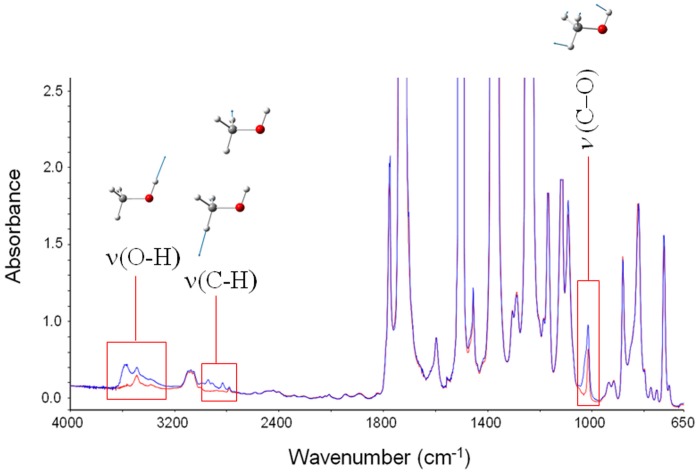
**Absorbance spectra for fully dried PMDA-ODA (red trace) and PMDA-ODA equilibrated at *p*/*p*_0_ = 0.5**. The insets represent the bands characteristic of sorbed methanol, and the forms of the respective normal modes.

In Figure 1 are reported the FTIR spectra of the fully dried polyimide film (red trace) and of the same film after equilibration with methanol vapor at *p*/*p*_0_ = 0.5 (blue trace). Sorbed methanol displays characteristic bands in three distinct regions of the spectrum, namely, in the 3650–3050 cm^−1^ range [ν (O-H)], in the 3000–2060 cm^−1^ range [ν (C-H)] and at around 1000 cm^−1^ (Shurvell, 2002). The latter peak is a highly coupled vibration, generally quoted as a ν (C-O), but with a significant contribution from the C-O-H bending. The methanol molecule represents an ideal probe to investigate the chemical environment surrounding the penetrant for it has one fragment very sensitive to H-bonding interactions (the hydroxyl group) and the other (the methyl group) completely insensitive. Thus, the ν (O-H) mode is expected to be the most perturbed, while the ν (C-H) vibrations should remain unaffected.

**Figure 2:**
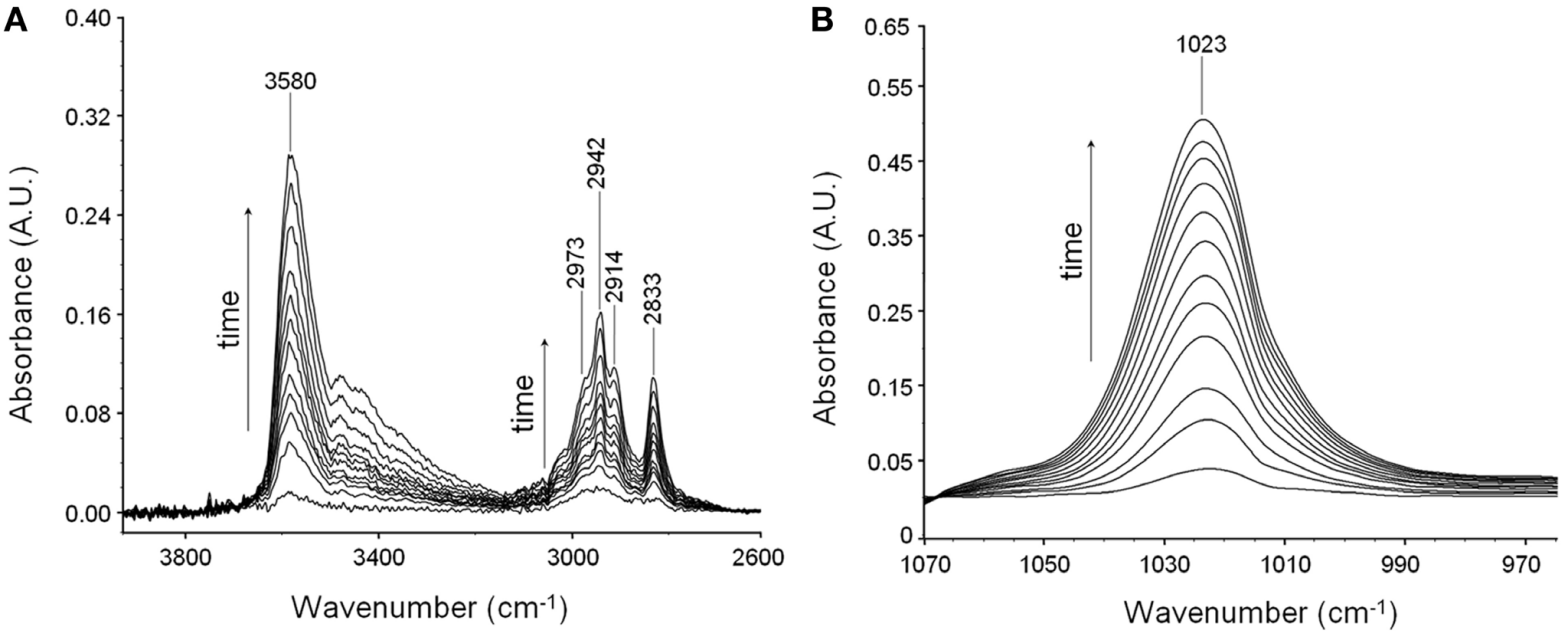
**Difference spectra collected at increasing times during a sorption experiments of methanol in PMDA-ODA at *p*/*p*_0_ = 0.3. (A)** Wavenumber range 4000–2700 cm^−1^. **(B)** Wavenumber range 1060–970 cm^−1^.

Suppressing the interference of the matrix by difference spectroscopy allows us to isolate the spectrum of sorbed methanol in the regions of interest. These are displayed in Figures 2A,B.

**Figure 3:**
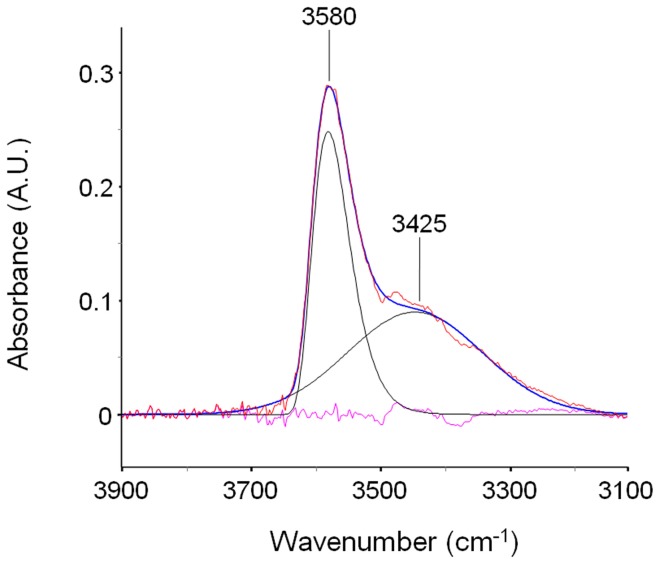
**LSCF analysis of the ν(*OH*) region (3900–3100 cm^−1^)**. The fitted spectrum is relative to a sample equilibrated in methanol vapor at *p*/*p*_0_ = 0.3. The figure displays the experimental profile (red trace), the best-fitting result (blue trace), the two resolved components (black traces) and the residual (cyan trace, experimental—best-fit).

In the ν (O-H) range a relatively sharp peak at 3585 cm^−1^ is superimposed onto a much broader band centered at lower wavenumbers. The band at 1023 cm^−1^ does not show evidence of an underlying fine structure. To get information about molecular interactions we analyzed in more detail the most sensitive region, i.e., the ν (O-H) interval which was subjected to a curve fitting analysis. The results obtained on the difference spectrum collected at equilibrium (*p*/*p*_0_ = 0.3) are shown in Figure 3. By properly selecting the band-shape of the peaks (a log-normal function and a mixed Guss-Lorentz function for the high- and low-frequency components, respectively) the experimental profile can be very satisfactorily simulated, which suggests the presence of two distinct molecular species.

**Figure 4:**
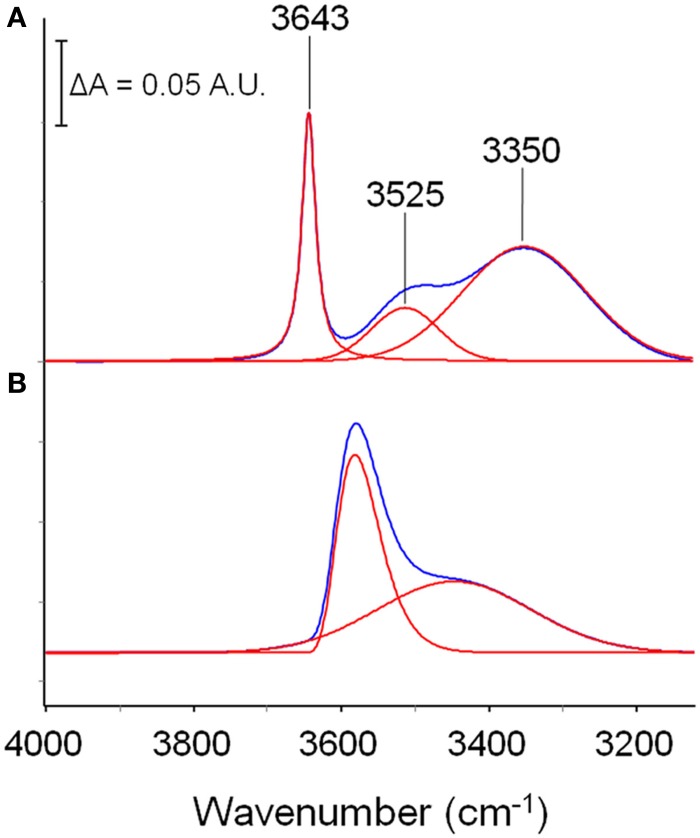
**Comparison between the spectrum of a methanol solution in CCl_4_ (0.10 M) and the spectrum of methanol sorbed in PMDA-ODA (*p*/*p*_0_ = 0.3)**. The absorbance scale refers to traces **(A)**; traces **(B)** have been expanded to full-scale to facilitate the comparison.

In order to propose a reasonable interpretation of the spectroscopic results, it is useful to compare the spectrum of methanol sorbed in the polyimide with that of methanol in unperturbed conditions. This reference state cannot be represented by the isolated molecule *in-vacuo* (i.e., in the gas phase, at low *p*) because of the superposition of roto-vibrational effects; a more appropriate reference is a dilute solution in a low polarity, non-interacting solvent (CCl_4_). In these conditions, at high dilution (below 0.01 M) only the monomer is present, giving rise to a very sharp peak at 3643 cm^−1^; increasing the concentration, dimers start to appear, producing a well resolved and symmetrical band at 3525 cm^−1^; At 0.1 M and above, the equilibrium involves also trimers and tetramers, which produce a further broad band centered at 3350 cm^−1^ (Dixon et al., 1997; Galizia et al., 2013). The spectrum of a 0.1 M solution of methanol in CCl_4_ is reported in Figure 4A. It is noteworthy the considerable increase of full width at half height (FWHH) when passing from the monomer to higher aggregates, which is a direct consequence of the H-bonding interaction in self-associated structures.

The spectrum of methanol sorbed in PMDA-ODA (*p*/*p*_0_ = 0.3) is shown in Figure 4B. By comparison with the reference state, the main peak at 3580 cm^−1^ can be safely associated with a monomeric species. However, the larger value of FWHH (from 22 to 90 cm^−1^) and, especially, the considerable red-shift (68 cm^−1^) demonstrate that in the polymer, the monomeric species is not free as in the CCl_4_ solution, but is involved in a H-bonding interaction of the non-self association type. Instead, self-association is related to the broad band at 3425 cm^−1^: its symmetrical shape suggests the occurrence of a single type of molecular aggregate (dimers) (Galizia et al., 2013) or, at least, a large prevalence of this species over higher order aggregates.

To identify the active site on the polymer backbone acting as proton acceptor, we ought to investigate the perturbation brought about by methanol to the spectrum of the polyimide. A thin film (in spectroscopic terms, i.e., less than 5.0 μm) is required to perform this analysis, in order to keep the most intense peaks within the limits of absorbance linearity. This sample was prepared *ad-hoc* by a spin-coating process (see experimental); the resulting thickness, as measured by the interference-fringes method (Musto et al., 2007) was 2.4 μm.

**Figure 5:**
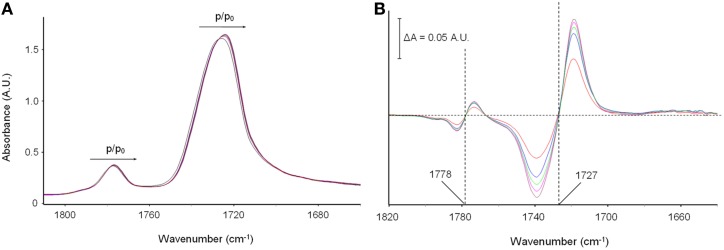
**(A)** The Absorbance spectra in the ν (C = O) frequency range (1810–1640 cm^−1^) collected on polyimide film equilibrated at different vapor pressures of methanol; **(B)** difference spectra (equilibrated sample–dry sample). The color code is as follows: black trace: *p*/*p*_0_ = 0 (dry sample); red trace: *p*/*p*_0_ = 0.2; blue trace: *p*/*p*_0_ = 0.3; green trace: *p*/*p*_0_ = 0.4; cyan trace: *p*/*p*_0_ = 0.5; brown trace: *p*/*p*_0_ = 0.6.

Figures 5A,B display the carbonyl range of the PMDA-ODA spectrum in the sample equilibrated at different relative pressures of methanol vapor. In particular, Figure 5A shows the absorbance spectra, while Figure 5B displays the difference spectra (equilibrated sample–dry sample).

It is observed that the two carbonyl peaks of the imide moiety [ν*_s_*(C = O) at 1778 cm^−1^ and ν_as_(C = O) at 1727 cm^−1^] are shifted toward lower frequencies. The effect is very weak in the absorbance spectra, yet well within the detectability limits of interferometric spectroscopy. It is further evidenced in the difference spectra where the typical first-derivative profiles characteristic of a downward shift of the sample peaks with respect to the reference, are readily apparent. The extent of the red-shift, which is reflected in the peak-to-peak height of the difference spectra, is found to increase gradually with increasing methanol concentration in the sample (see Figure 5B). It has been verified that the effect is fully reversible upon methanol removal, which further confirms that it originates from the polyimide/methanol interactions.

The above observations can be interpreted assuming that the carbonyl groups of the polyimide act as proton acceptors in the H-bonding interaction with methanol. In fact, the red shift is a direct consequence of the weakening of the C = O force constant due to the redistribution of the electron density caused by the interacting proton.

**Scheme 2:**
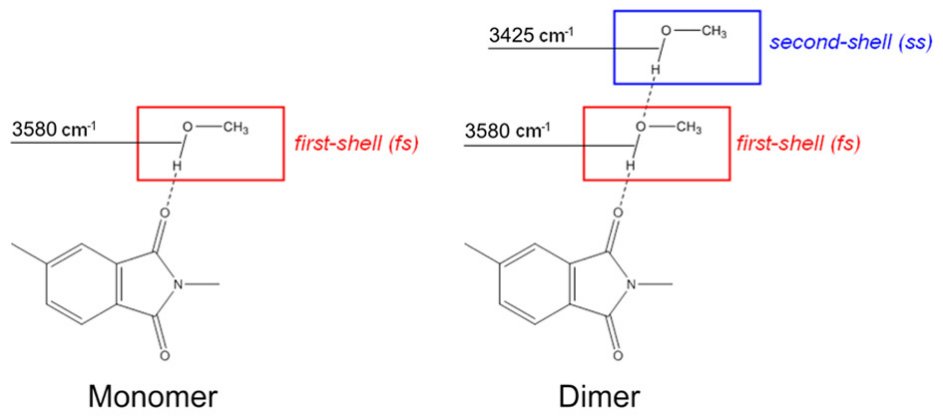
**Schematic representation of the methanol species identified by the spectroscopic analysis**.

Taking into consideration the whole of the spectroscopic results, we can assign the 3580 cm^−1^ component to the O-H groups of methanol directly bound to the imide carbonyls, which represent the first shell layer of sorbed methanol in the frame of the multilayer adsorption model of Brunauer-Emmett-Teller (BET) [19]. The band at 3425 cm^−1^ originates from self-associated methanol molecules, as depicted in Scheme 2.

Work is in progress to optimize the geometry of the above molecular aggregates by first *principles* computational approaches so as to calculate theoretically the vibrational spectra and compare these simulations with the experimental results.

### Diffusion behavior

**Figure 6:**
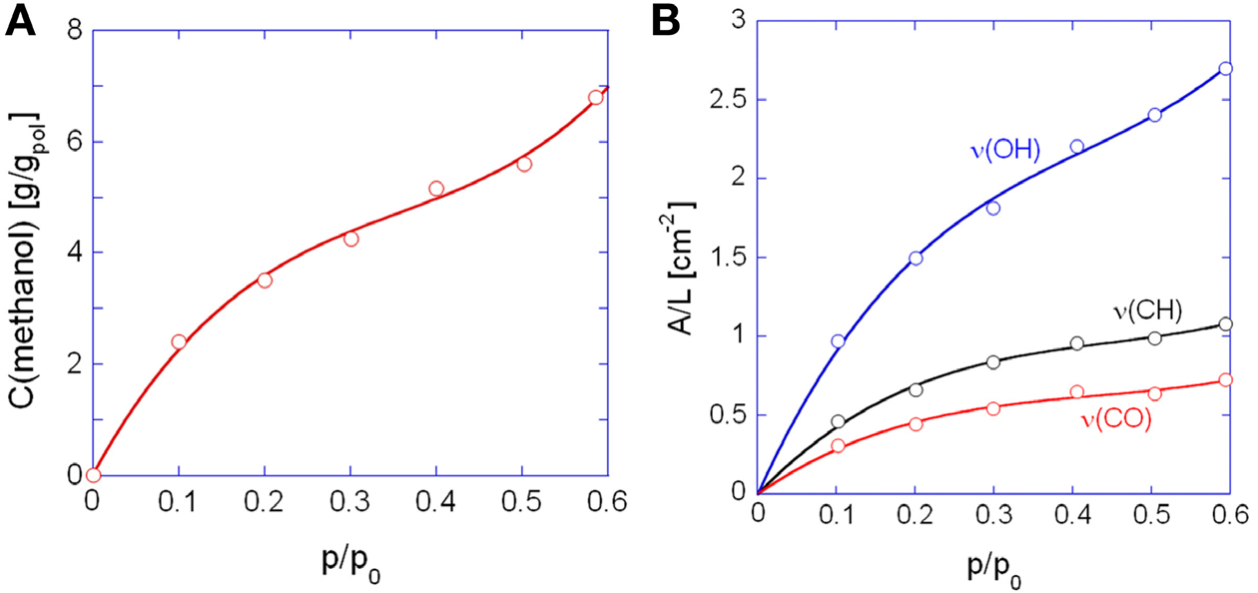
**Sorption isotherms as a function of the relative pressure of methanol. (A)** Gravimetric measurements; **(B)** spectroscopic measurements. In **(B)** are reported the curves obtained with the indicated bands of methanol. Solid lines are for eye guidance only.

In Figure 6A is reported the sorption isotherm as a function of the relative pressure of methanol, as evaluated gravimetrically. S-shaped curves similar to those of Figure 6A have been reported in the literature for methanol and other alkyl-alcohols absorbed in high *T_g_*, glassy polyacetylenes (Galizia et al., 2011, 2012). In the case at hand, this behavior is likely due to a dual-mode diffusion regime. The upturn present in sorption isotherms has been associated with two different factors: (1) plasticization of the glassy matrix induced by the penetrant, which promotes the transition from a glassy to a rubbery system, and (2) the gradual onset of penetrant clustering or self-association as the relative pressure increases. In view of the high glass transition temperature of the polyimide (383°C) the second interpretation seems the most likely. The solubility of methanol in PMDA-ODA is conspicuous, close to 7.0 wt% at *p*/*p*_0_ = 0.6, which reflects both the favorable H-bonding interactions and the contribution of the methyl group in reducing the alcohol polarity.

**Figure 7:**
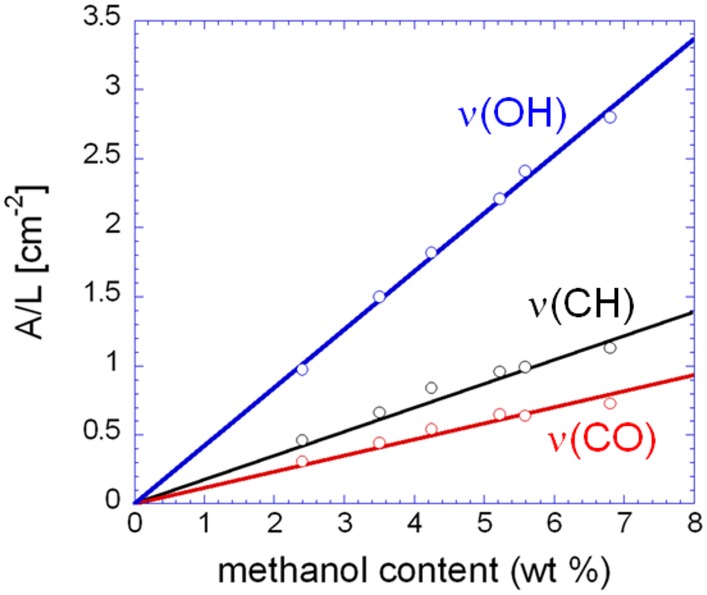
**Normalized absorbance of the three analytical bands of methanol as a function of the methanol concentration in the sample**.

In Figure 6B are shown the isotherms evaluated spectroscopically by considering the absorbance areas (A) normalized for the sample thickness (L) of the three analytical bands of the penetrant. The absorbance-concentration relationships are demonstrated in Figure 7: in all cases a Lambert-Beer behavior is observed (i.e., a linear trend through the origin) which allows us to directly transform the absorbance intensities into absolute concentration values.

**Figure 8:**
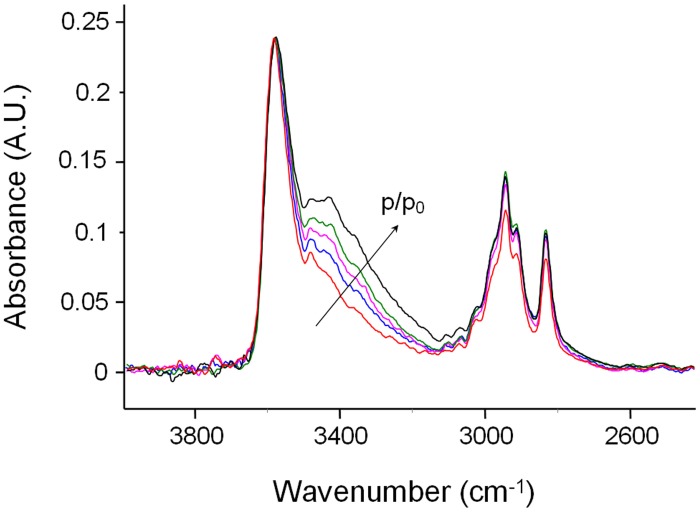
**Difference spectra in the ν(OH)—ν(CH) region (4000–2400 cm^−1^) of PMDA-ODA films equilibrated at different relative pressures of methanol vapor**. Color code: red trace: *p*/*p*_0_ = 0.2; blue trace: *p*/*p*_0_ = 0.3; cyan trace: *p*/*p*_0_ = 0.4; green trace: *p*/*p*_0_ = 0.5; black trace: *p*/*p*_0_ = 0.6. The absorbance scale refers to the red trace; the other spectra were normalized with respect to the peak at 3580 cm^−1^ to facilitate the comparison.

Figure 8 shows the difference spectra representative of sorbed methanol at the various investigated relative pressures. Normalization with respect to the principal component at 3580 cm^−1^, evidences that the contribution of the component at 3425 cm^−1^ to the total absorbance area of the ν (OH) profile increases by increasing *p*/*p*_0_, that is, by enhancing the concentration of sorbed methanol. This indicates that, at higher concentration of sorbed methanol the equilibrium moves toward self-associated species. To put this observation on a more quantitative basis, we performed a LSCF analysis on the spectral profiles of Figure 8; the quality of the fit was in all cases satisfactory, analogous to that reported in Figure 3. The absorbance of the curve-resolved components may be converted into concentration values provided that the respective values of molar absorptivity (ε*_fs_*, ε*_ss_*) are available. In a recent publication on the sorption of methanol in a commercial polyetherimide (Ultem 1000) analogous spectral features were observed and a method was proposed to evaluate ε*_fs_* and ε*_ss_* based on coupling spectroscopic and gravimetric measurements taken at identical equilibrium conditions (Galizia et al., 2013). The reported values were 76 km/mol for ε*_fs_* and 108 km/mol for ε*_ss_*. Assuming the above values for the system PMDA-ODA/methanol, we estimated *C_fs_* and *C_ss_* from the relevant absorbance–concentration relationships, i.e.,*C_fs_* = *A*_3575_/ε_*fs*_*L* and *C_ss_* = *A*_3440_ /ε_*ss*_ *L*. The results of the quantitative analysis are summarized in Table 1.

**Table 1:** **Sample thickness, absorbance of the resolved components, concentration of methanol species and total methanol concentration for the sorption tests performed at different relative pressures of methanol vapor**.

**p/p_0_**	***L* (cm)**	***A_3580_* (cm^−1^)**	***A_3425_* (cm^−1^)**	***C_fs_* (mmol/cm^3^)**	***C_ss_* (mmol/cm^3^)**	**C^spec^_tot_ (mmol/cm^3^)**	**C^grav^_tot_(mmol/cm^3^)**
0.2	2.4 × 10^−3^	14.37	14.79	0.84	0.61	1.45	2.70
0.3	2.2 × 10^−3^	18.92	21.56	1.03	0.83	1.86	3.29
0.4	2.4 × 10^−3^	18.47	25.23	1.22	1.17	2.39	4.05
0.5	2.0 × 10^−3^	20.13	31.27	1.30	1.42	2.72	4.33
0.6	2.0 × 10^−3^	23.60	47.14	1.29	1.79	3.08	5.15

**Figure 9:**
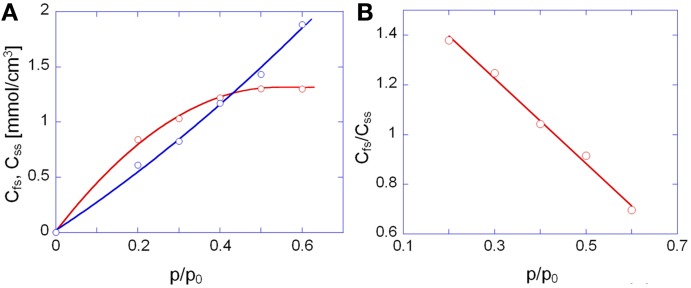
**(A)** Concentration of first-shell (red circles) and second-shell (blue circles) methanol as a function of *p*/*p*_0_. **(B)** Concentration ratio of first-shell to second-shell species as a function of *p*/*p*_0_. In **(A)** the continuous lines are a guide for the eye.

In Figure 9A are reported the concentrations of *firs-shell* and *second- (or higher-) shell* methanol in the polyimide as a function of methanol relative pressure. The *C_fs_* curve exceeds the *C_ss_* curve up to a *p*/*p*_0_ value of 0.4; in this range it is likely that dimers are the prevailing species, with a slight amount of residual monomers, whose concentration is equal to *C_fs_* – *C_ss_*. At *p*/*p*_0_ values higher than 0.4 *C_ss_* significantly offsets *C_fs_* and this may only occur when aggregates comprising more than two methanol molecules (clusters) are formed. The plot of Figure 9A allows us to clearly identify the onset of the clustering phenomenon as the intersection point of the two curves. It is noted that that the absolute spectroscopic evaluation of sorbed methanol as *C_fs_* + *C_ss_*, (that is, with no direct calibration with gravimetry), provides concentration values of the same order of magnitude as those from weight measurements but underestimated by a factor of about 1.6. This could be due to the assumed absorptivity values which may slightly differ in going from a polyetherimide to the PMDA-ODA. Figure 9B demonstrates that the concentration ratio *C_fs_*/*C_ss_* decreases linearly by increasing the relative pressure of methanol vapor.

**Figure 10:**
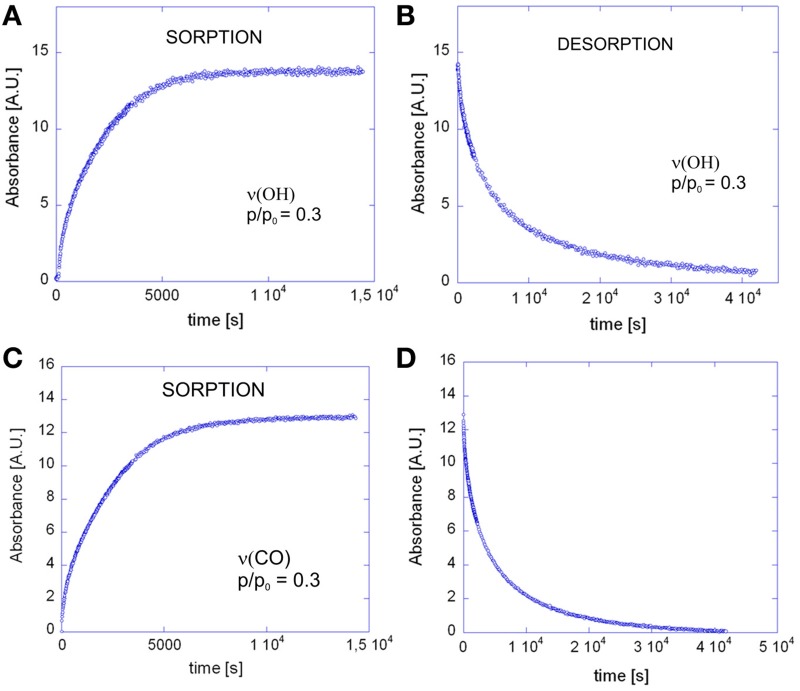
**Absorbance of the ν(OH) band of methanol as a function of time for: (A) the sorption test at *p*/*p*_0_ = 0.3; (B) the desorption test**. Absorbance of the ν(CO) band of methanol as a function of time for: **(C)** the sorption test at *p*/*p*_0_ = 0.3; **(D)** the desorption test.

The evolution of the infrared spectrum with time (see Figures 2A,B) can be reliably used to trace the kinetics of the mass transport, both in the sorption and in the desorption regime. This is demonstrated in Figures 10A–D, which display the absorbance versus time curves relative to the ν (OH) and the ν (CO) bands.

The above data have been analyzed on the basis of the differential equation representing the Fick's second law of diffusion, imposing the initial condition (I.C.) and the boundary conditions (B.C.'s) appropriate for the case of a plane sheet exposed to equal penetrant activity on both surfaces. In terms of concentration of penetrant within the polymer, the I.C. and B.C.'s are as follows:

$$\begin{array}{l} I.C.:\;C=C_{0}\;0<x<L,\;t=0\\ B.C.^{\prime }s:\;C=C_{1}\text{ at }x=0,\;\forall t\geq 0\\ \;C=C_{1}\text{ at }x=L,\;\forall t\geq 0 \end{array}$$

where *L* represents the thickness of the plane sheet. For this configuration, the solution of the second Fick's law is (Crank, 1975):

(3) $$\frac{m(t)}{m_{\inf}}=1-\frac{8}{\pi^{2}}\sum_{m=0}^{\infty }\frac{1}{{\left(2m+1\right)}^{2}}\exp\text{ ​}\left[\frac{-D{\left(2m+1\right)}^{2}\pi^{2}t}{L^{2}}\right]$$

where *m*(*t*) and *m_inf_* are, respectively, the total mass of penetrant absorbed within the sheet at time *t* and at sorption equilibrium while *D* is the mutual diffusivity.

Equivalently, in terms of absorbance, the solution reads:

(4) $$\frac{A(t)}{A_{\inf}}=1-\frac{8}{\pi^{2}}\sum_{m=0}^{\infty }\frac{1}{{\left(2m+1\right)}^{2}}\;\exp\;\left[\frac{-D{\left(2m+1\right)}^{2}\pi^{2}t}{L^{2}}\right]$$

where *A*(*t*) and *A_inf_* are the integrated absorbances at time *t* and at equilibrium, respectively.

**Figure 11:**
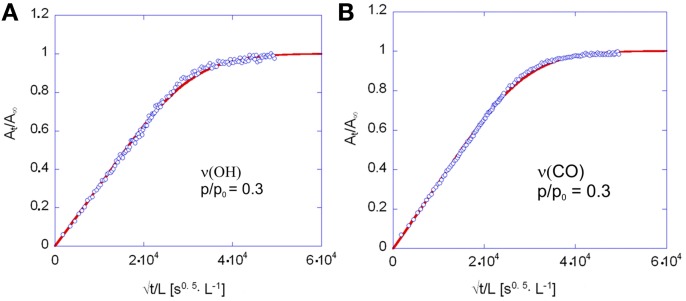
**Fick's diagrams (i.e., *A_t_*/*A*_*inf*_ vs. √*t*/*L*) obtained from the data of Figure 10A (A) and Figure 10C (B)**. Open circles represent experimental data, solid lines are the least-squared best-fitting of the data points with Equation 4. The diffusion coefficient, *D* in Equation 4 was the sole parameter allowed to change in the fitting process.

The coincidence of the experimental data with the curves predicted by theory, as well as the linearity of the sorption curves as a function of *t*^0.5^/*L*, up to *A*(*t*)/*A*_*inf*_ values well exceeding 0.6 (see Figures 11A,B) demonstrates the Fickian behavior of the system.

**Figure 12:**
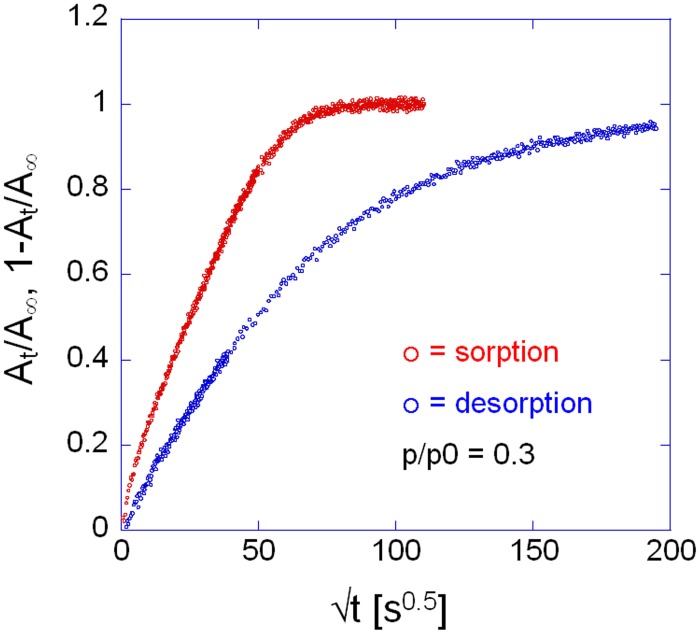
**Comparison between sorption and desorption kinetics as represented in Fick's diagrams**. Measurement performed at *p*/*p*_0_ = 0.3, via time-resolved FTIR spectroscopy. Analytical band: ν(CO) at 1023 cm^−1^.

**Figure 13:**
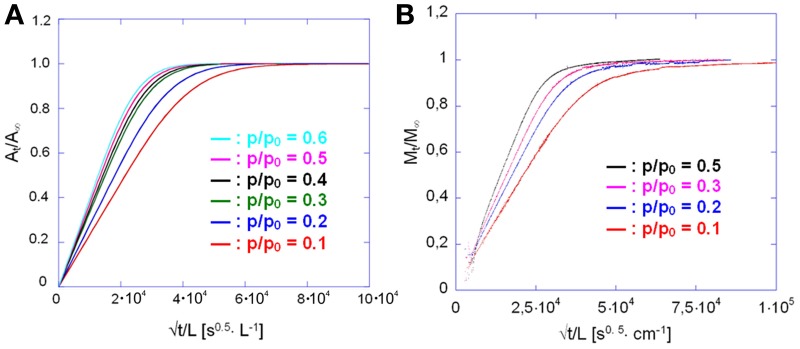
**Sorption kinetics of methanol in PMDA-ODA at different relative pressures, as indicated. (A)** Spectroscopic measurements; **(B)** gravimetric measurements.

Comparing the sorption and desorption kinetics (Figure 12) evidences a much slower diffusivity in desorption than in sorption, which is typical of systems characterized by a pronounced dependence of *D* on the total concentration of penetrant in the sample. In particular, the observation that the desorption curve lies well below the sorption curve, implies that *D* is an increasing function of concentration. To investigate further this effect the kinetic analysis of the sorption process was performed in the whole range of relative pressures of methanol from 0.1 to 0.6. The results of both the spectroscopic and the gravimetric measurements are reported in Figures 13A,B.

The diffusion coefficient can be evaluated directly from Equations 3 or 4 whenever it can be considered constant with penetrant concentration. If this is not the case, a slightly more elaborate procedure is applied (Crank, 1975). The diffusivity from the Fick's plot represents now an average value over the whole range of concentrations encompassed in the diffusion process, i.e.,

(5) $$\bar{D}=\frac{1}{C_{0}}\int_{0}^{C_{0}}DdC$$

where *C*_0_ is the methanol concentration at equilibrium in a specific sorption test. Thus, the *D* values from Equations 3 or 4 are plotted as *DC*_0_ vs. *C*_0_ (Equation 5) and numerical differentiation of the curve with respect to *C*_0_ yields the sought *D* vs. *C* relationship.

The results of such an analysis are summarized in Table 2.

**Table 2:** **Sample thickness, equilibrium methanol content and diffusivities for the sorption tests performed at different relative pressures of methanol vapor**.

**p/p_0_**	**L_spec_ (cm)**	**C_0_ (wt %)**	**D_spec_ (cm^2^/s)**	**L_grav_ (cm)**	**D_grav_ (cm^2^/s)**	**D(C)(cm^2^/s)**
0.1	2.4 × 10^−3^	2.40	1.06 × 10^−10^	1.6 × 10^−3^	1.10 × 10^−10^	2.34 × 10^−10^
0.2	2.2 × 10^−3^	3.51	1.51 × 10^−10^	1.5 × 10^−3^	1.57 × 10^−10^	3.30 × 10^−10^
0.3	2.4 × 10^−3^	4.25	2.19 × 10^−10^	1.5 × 10^−3^	1.96 × 10^−10^	3.94 × 10^−10^
0.4	2.0 × 10^−3^	5.23	2.41 × 10^−10^	1.6 × 10^−3^	2.21 × 10^−10^	4.79 × 10^−10^
0.5	2.0 × 10^−3^	5.59	2.73 × 10^−10^	2.0 × 10^−3^	2.54 × 10^−10^	5.10 × 10^−10^
0.6	2.4 × 10^−3^	6.77	3.04 × 10^−10^	1.3 × 10^−3^	3.19 × 10^−10^	6.12 × 10^−10^

**Figure 14:**
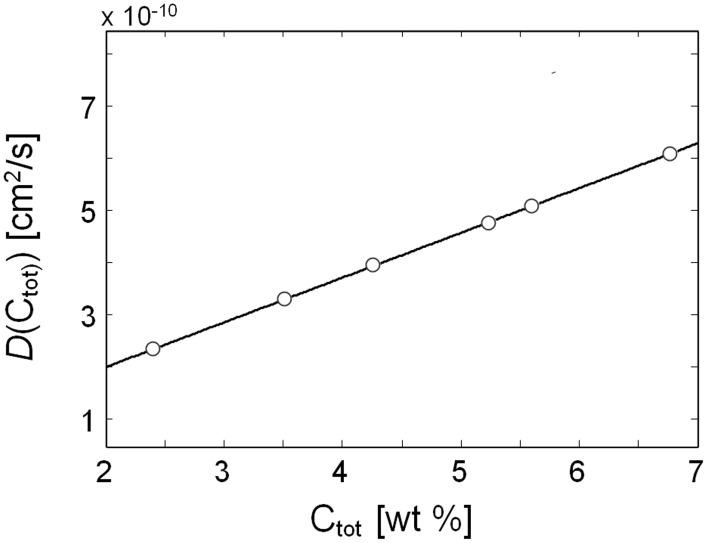
**Diffusivity of methanol vapor in PMDA-ODA as a function of the total concentration of methanol in the sample**.

From Table 2 it emerges that the diffusivity values estimated from gravimetric and FTIR data are in excellent agreement with each other; moreover, they also compare well with a previous literature report (Kamaruddin and Koros, 2000). The plot of *D* vs. *C*, constructed considering the average between the spectroscopic and the gravimetric values of *D*, reveals a linear relationship in the concentration range of interest.(see Figure 14).

## Conclusions

In this work the sorption of methanol in PMDA-ODA polyimide has been investigated both kinetically and at equilibrium, coupling gravimetric and *in-situ* FTIR spectroscopy in the transmission mode. This approach allowed a detailed characterization of the investigated system at a molecular level.

The spectral data at equilibrium were analyzed by using two complementary techniques, i.e., difference spectroscopy and least squared curve-fitting analysis: two distinct molecular species were detected in the system: single methanol molecules directly bound to the carbonyls of the imide group via H-bonding (*first-shell* species), and self-associated methanol molecules forming second- and higher shell layers. Up to *p*/*p*_0_ values of 0.4 the dimer is likely to represent the predominant species; afterwards, higher aggregates (higher shell layers) are formed, giving rise to the clustering phenomenon. A method was proposed to quantify the population of the methanol species, based on literature values of the respective molar absorptivities. The concentration ratio *C_fs_*/*C*_*ss*_ was found to decrease linearly by increasing the relative pressure of methanol vapor.

Kinetic data from gravimetry and *time-resolved* spectroscopy provided two independent estimations of the diffusion coefficients which were in good agreement with each other. The system was found to behave according to the Fick's second law of diffusion, with the diffusivity displaying a marked dependence on the total concentration of sorbed methanol. The evaluation of the *D* vs. *C* curve with the differential method of Crank demonstrated a linear behavior with positive slope.

### Conflict of interest statement

The authors declare that the research was conducted in the absence of any commercial or financial relationships that could be construed as a potential conflict of interest.
